# Population structure, antibiotic resistance and molecular characteristics of Streptococcus pneumoniae causing invasive disease in Hubei, China

**DOI:** 10.1099/jmm.0.002015

**Published:** 2025-05-16

**Authors:** Jiangqin Song, Huan Zhang, Siyu He, Huabing Yuan, Yunbo Chen

**Affiliations:** 1 Department of Laboratory, The First People’s Hospital of Tianmen City (Tianmen Hospital Affiliated to Wuhan University of Science and Technology), Tianmen, Hubei 431700, PR China; 2 Hubei Province Key Laboratory of Occupational Hazard Identification and Control, Wuhan University of Science and Technology, Wuhan, Hubei 430070, PR China; 3 Department of Pharmacy, The First People’s Hospital of Tianmen City (Tianmen Hospital Affiliated to Wuhan University of Science and Technology), Tianmen, Hubei 431700, PR China; 4 State Key Laboratory for Diagnosis and Treatment of Infectious Diseases, Collaborative Innovation Center for Diagnosis and Treatment of Infectious Diseases, The First Affiliated Hospital, School of Medicine, Zhejiang University, Hangzhou, Zhejiang 310003, PR China

**Keywords:** antibiotic resistance, multilocus sequence typing (MLST), serotype, *Streptococcus pneumoniae*, whole-genome sequencing

## Abstract

**Introduction.** This research sought to examine the epidemiological features, antibiotic resistance profiles and molecular characteristics of *Streptococcus pneumoniae* infections among hospitalized patients in Hubei, China, thus providing epidemiological evidence to inform the effective prevention and management of pneumococcal infections.

**Hypothesis/Gap Statement.** Current research predominantly focuses on large urban centres, leaving a substantial knowledge gap regarding pneumococcal infections and resistance patterns in smaller city-level hospitals, such as those in Hubei Province.

**Aim.** This study aimed to investigate the epidemiological features, antibiotic resistance profiles and molecular characteristics of invasive *S. pneumoniae* isolates from hospitalized patients in Tianmen City, Hubei, China.

**Methodology.***S. pneumoniae* strains were isolated from hospitalized patients at the First People’s Hospital of Tianmen, Hubei, China, between September 2021 and September 2022. Epidemiological characteristics, serological typing and antimicrobial susceptibility of the isolates were analysed. Whole-genome sequencing was performed on the strains to identify resistance genes and virulence genes.

**Results.** A total of 194 *S*. *pneumoniae* isolates were analysed, with 90% (174/194) from community-acquired pneumonia cases. Respiratory samples accounted for 96% of isolates, and children under 5 years old comprised 78% of cases. Seasonal variation was observed, with infections peaking in winter and spring. Antibiotic resistance analysis revealed notable age-related differences: penicillin resistance was 12.37% overall but absent in elderly patients. Ceftriaxone showed no resistance, whereas cefotaxime and meropenem exhibited higher resistance in children than in the elderly. Multilocus sequence typing identified 56 sequence types (STs), with ST271 (24.2%) being the most prevalent. Serotyping revealed 24 serotypes, with 19F (27.8%, 54/194) as the dominant type. Phylogenetic analysis showed two major clades, with strong correlations between serotype and ST distribution. Resistance genes *ermB* and *tetM* were highly prevalent (99.0% and 97.9%, respectively). Comparative genomic analysis demonstrated significantly higher resistance rates in ST271 strains than in non-ST271 strains, particularly for cefotaxime (76.60% vs. 4.76%) and meropenem (53.19% vs. 14.29%). ST271 strains predominantly expressed serotype 19F, accounting for 87% (47/54) of all serotype 19F isolates, carrying distinct resistance and virulence genes, highlighting its clinical significance.

**Conclusion.** This study highlights a significant burden of invasive *S. pneumoniae* infections, predominantly affecting children under five, with notable peaks during winter and spring. ST271, predominantly associated with serotype 19F, exhibited significantly higher antibiotic resistance rates compared with other strains, indicating the necessity of tailored antibiotic strategies and robust local antibiotic stewardship programmes. The widespread presence of resistance and virulence genes underscores the evolutionary adaptability of *S. pneumoniae*, emphasizing the importance of continuous genetic surveillance. The current pneumococcal vaccination (13-valent pneumococcal conjugate vaccine) coverage of the predominant serotype provides a favourable outlook for disease control.

**Impact Statement**

This study highlights significant antibiotic resistance and genetic diversity of invasive *Streptococcus pneumoniae* isolates from a previously underreported region, informing regional antibiotic stewardship and vaccination policies.

## Data Summary

The whole-genome sequencing data of 194 *Streptococcus pneumoniae* isolates generated in this study are available in the NCBI database under BioProject accession number PRJNA1236896.

## Data Availability

The original contributions presented in this study are included in the article. Further inquiries can be directed to the corresponding authors.

## Introduction

*Streptococcus pneumoniae* is a significant global pathogen responsible for a range of invasive diseases, including pneumonia, otitis media, sepsis and meningitis, which are often associated with severe complications and high mortality rates [1]. In China, *S. pneumoniae* is one of the leading causes of morbidity and mortality, particularly among children and the elderly [2]. *S. pneumoniae* comprises numerous serotypes, all of which possess pathogenic potential. The most prevalent serotypes include 14, 19F, 3, 6A, 6B, 18C, 19A, 9V and 23F, collectively responsible for over 70% of all cases [3]. Although vaccination rates with the 13-valent pneumococcal conjugate vaccine (PCV13) have been increasing among children, the relatively high cost of the vaccine and its limited coverage under insurance schemes have resulted in significant regional disparities in vaccination rates, especially in rural and less developed areas [4,5]. Antibiotics remain the cornerstone of treatment for pneumococcal infections. However, the global rise in antibiotic resistance has become a growing concern. In response, the World Health Organization (WHO) has classified penicillin-resistant *S. pneumoniae* (PRSP) as a priority pathogen [6,7].

China’s large population, high population density and substantial proportion of children and elderly individuals create favourable conditions for the spread of *S. pneumoniae*. However, due to the challenges associated with isolating the pathogen, empirical antibiotic treatment is widely adopted in clinical practice. Compared with data from developed countries, antibiotic resistance rates in *S. pneumoniae* are generally higher in Mainland China [6]. For instance, a study on antibiotic resistance in *S. pneumoniae* isolated from children in Chongqing reported resistance rates of 63.9% to azithromycin, 56.6% to amoxicillin, 41% to rifampin, 37.3% to amoxicillin-clavulanate, 37.3% to trimethoprim-sulfamethoxazole and 3.6% to ceftriaxone [8]. Similarly, among adults in Shanghai, resistance rates were found to be 92.00% to erythromycin, 90.67% to azithromycin, 86.67% to tetracycline, 81.33% to clindamycin, 54.67% to cefaclor and 54.67% to trimethoprim-sulfamethoxazole, with 78.67% of strains exhibiting multidrug resistance [9]. These data highlight significant regional and age-related variations in resistance patterns across China. Consequently, analysing the antibiotic resistance profiles of *S. pneumoniae* in specific regions is crucial for reducing the burden of invasive infections and guiding effective clinical treatment strategies. Such analyses also provide insights into potential new antimicrobial drug targets and mechanisms of action.

Current studies on *S. pneumoniae* resistance in Mainland China have primarily focused on large urban hospitals [8,9], with limited attention given to smaller city hospitals. A previous study conducted in Jingzhou City, Hubei Province, highlighted the burden of invasive pneumococcal disease (IPD) and identified serotypes 14, 12 and 19F as the predominant strains among hospitalized patients [10]. This study emphasized the urgent need for enhanced vaccination coverage and improved antibiotic stewardship [10]. However, research remains scarce in smaller cities and rural areas of Hubei Province, particularly regarding the molecular epidemiology, resistance patterns and virulence profiles of *S. pneumoniae*. This study aims to perform a retrospective analysis of *S. pneumoniae* infections in hospitalized patients over 1 year at a city-level hospital. We will investigate the resistance patterns and serotype distribution in this region and integrate these findings with whole-genome sequencing (WGS) to analyse resistance genes, virulence genes and other molecular characteristics. Our findings are expected to provide critical data for guiding empirical treatment strategies, informing vaccine development, identifying new antimicrobial drug targets and advancing the understanding of drug resistance mechanisms.

## Methods

### Definition

According to the American Thoracic Society (ATS) and the Infectious Diseases Society of America (IDSA), community-acquired pneumonia (CAP) refers to an acute infection of the pulmonary parenchyma acquired outside of healthcare settings. It occurs in individuals who have not been recently hospitalized or had significant exposure to the healthcare system. Hospital-acquired pneumonia (HAP) is defined as pneumonia that occurs 48 h or more after hospital admission and is not incubating at the time of admission. HAP carries higher morbidity and mortality risks due to the increased prevalence of multidrug-resistant pathogens.

### Clinical strain isolation and information collection

Clinical specimens (including sputum, blood and bronchoalveolar lavage) were collected from patients treated at the First People’s Hospital of Tianmen, Hubei Province, between 1 September 2021 and 31 August 2022, for the isolation of *S. pneumoniae*. Repeated isolates from the same patient and the same anatomical site were excluded, with only the first isolate retained for analysis. Tianmen is located in the central-southern part of Hubei Province, covering both urban and rural areas.

Specimens were collected on the day of hospitalization or during the first visit, including sputum, bronchoalveolar lavage and blood. Respiratory specimens were processed following the *Guidelines for Bacterial Culture of Lower Respiratory Tract Infections* (WS/T 499-2017), in accordance with the *National Clinical Laboratory Guidelines* (fourth edition) [11]. Sputum samples were subjected to Gram staining, and only specimens with <10 epithelial cells and >25 white blood cells per low-power field were considered suitable for culture. For children under 5 years old who are unable to expectorate sputum spontaneously, nasopharyngeal aspirates or induced sputum were collected.

Specimens were inoculated onto 5% sheep blood agar plates (Zhengzhou Autobio Biotechnology Co., Ltd., China) and chocolate agar plates (Zhengzhou Autobio Biotechnology Co., Ltd.), followed by incubation at 37 °C in a 5% CO_2_ atmosphere for 24–48 h. Colonies exhibiting typical *S. pneumoniae* morphology (*α*-haemolysis and mucoid appearance) were selected for further analysis. Presumptive *S. pneumoniae* isolates were confirmed using biochemical tests, including catalase (negative), optochin susceptibility (positive) and bile solubility (positive). Additionally, isolates were identified using matrix-assisted laser desorption ionization time-of-flight MS (VITEK-MS, bioMérieux, France).

Relevant patient information (gender, age, seasonal distribution, specimen source and clinical diagnosis) was recorded for each isolate. All strains were stored in Microbank (Pro-Lab Diagnostics, Canada) at −80 °C. Strains were subsequently transferred to the State Key Laboratory for Diagnosis and Treatment of Infectious Diseases, the First Affiliated Hospital, Zhejiang University, for strain revival and re-identification.

### *In vitro* antimicrobial susceptibility test

Antimicrobial susceptibility testing was performed using the VITEK-2 Compact system (bioMérieux), with susceptibility cards (GP68) from bioMérieux. The MIC for penicillin was determined using E-test strips, with penicillin E-test strips (Zhengzhou Autobio Biotechnology Co., Ltd.). The interpretation of the results followed the 2024 Clinical and Laboratory Standards Institute (CLSI) guidelines (M100-S34) [12].

Quality control was performed following the CLSI recommendations, using *Staphylococcus aureus* ATCC 29213 and *S. pneumoniae* ATCC 49619 as control strains. These strains were routinely maintained in the laboratory.

### DNA extraction and WGS

#### DNA extraction from bacterial strains

Genomic DNA was extracted using the DNeasy Blood and Tissue Kit (Qiagen, Germany) according to the manufacturer’s spin-column protocol. The quality of the DNA was assessed by NanoDrop spectrophotometry with A260/A280 ratios between 1.8 and 2.0 and A260/A230 ratios above 2.0.

#### Whole-genome sequencing

Genomic DNA was sequenced using the Illumina NovaSeq 6000 platform (Illumina, San Diego, CA, USA). DNA libraries were prepared using the Illumina DNA Prep kit following the manufacturer’s protocol, reaching a sequencing coverage of 200×. The sequence data were processed and quality controlled according to a standard pipeline. Following the preprocessing steps, assembly of the high-quality trimmed reads was accomplished using SPAdes (v.3.6, developed by Bankevich), with a scaffold N50 length of ~14.78 kb and the GC content of ~39.72% [13]. Assembly quality was assessed using QUAST (v5.2.0).

The WGS data of 194 *S*. *pneumoniae* isolates have been deposited into the National Center for Biotechnology Information (NCBI) public database under BioProject accession number PRJNA1236896.

#### Phylogenetic tree construction

Gene annotation was performed using Prokka v1.124 [14]. To investigate population structure, a maximum-likelihood phylogenetic tree was constructed based on core-genome alignments obtained in Roary using mega 11 with 1,000 bootstrap replicates and visualized using the Interactive Tree of Life web server [15,16].

### Multilocus sequence typing

Multilocus sequence typing (MLST) alleles and sequence types (STs) were identified *in silico* and queried against the PubMLST *S. pneumoniae* MLST database (https://pubmlst.org/spneumoniae/). An unknown ST was defined as an ST that was not present in the *S. pneumoniae* MLST database available on PubMLST.

### Identification of antimicrobial resistance genes and virulence genes

To investigate the genetic characteristics of *S. pneumoniae*, antimicrobial resistance (AMR) genes were detected using the Comprehensive Antibiotic Resistance Database (CARD) Resistance Gene Identifier (RGI) software (https://card.mcmaster.ca/analyze/rgi). Virulence genes were identified using the virulence factor database (VFDB) server (http://www.mgc.ac.cn/VFs/). Heatmap analysis was performed using R.

### Statistical methods

AMR analysis was performed using WHONET 5.6 software, and statistical analysis was conducted using SPSS 24.0 software. The significance of quantitative data was evaluated using t-tests, with *P*<0.05 considered statistically significant. Clinical characteristics were analysed using Excel.

## Results

### Clinical data of patients with isolated strains

A total of 194 patients with *S. pneumoniae* infections were included in this study. As summarized in Table 1, 90% (174/194) of the cases were from patients diagnosed with CAP, highlighting the significant burden of *S. pneumoniae* in CAP cases within the study population. Respiratory samples were the predominant sample type, accounting for ~96% (186/194) of all samples, while blood samples accounted for the remaining 4% (8/194).

**Table 1.** Clinical characteristics of 194 patients infected with *S. pneumoniae*

Characteristic	No. of patients	Percentage (%)
Total	194	100
Site of infection acquisition		
CAP	174	89.7
HAP	20	10.3
Gender		
Male	127	65.5
Female	67	34.5
Age (years)		
The mean patient age(sd)	13.78 (25.66)	
≤5	151	77.9
5–17	8	4.1
18–60	7	3.6
>60	28	14.4
Season		
Spring	74	38.1
Summer	22	11.4
Autumn	33	17.0
Winter	65	33.5
Specimen type from		
Sputum	185	95.4
Blood	8	4.1
Bronchoalveolar lavage	1	0.5
Length of stay (days: median and IQR)	6 (7.4)	–

– : Not applicable.

CAP, community-acquired pneumonia; HAP, hospital-acquired pneumonia.

Gender distribution analysis revealed a higher prevalence of *S. pneumoniae* infections among male patients with 127 cases (65.46%), compared with 67 cases (34.54%) in female patients. This suggests that males may be at an increased risk of developing *S. pneumoniae* infections, although further studies are needed to explore the underlying factors contributing to this observation.

Age distribution analysis demonstrated that children under 5 years old constituted the largest proportion of cases, representing ~78% of the study population. This finding aligns with global trends, where young children are recognized as a high-risk group for pneumococcal infections. Elderly patients aged over 60 accounted for 14%of cases, while individuals aged 5–60 years represented a smaller proportion (~8%). These results underscore the importance of targeted vaccination strategies and clinical interventions for both paediatric and elderly populations.

Seasonal analysis revealed a distinct pattern, with 72% of infections occurring during the spring and winter months. This seasonal clustering may be attributed to factors such as increased transmission rates during colder months, higher prevalence of respiratory viruses or seasonal changes in host immunity.

### AMR analysis of isolated strains

Significant differences in antibiotic susceptibility were observed across different age groups, reflecting age-related patterns of antibiotic resistance (Table 2). Overall, the resistance rate to penicillin was 12.37% (based on non-meningitis infection breakpoint criteria) with notable variations between age groups. Resistance was higher in children under 5 years old (12.58%), while no resistance was observed in elderly patients aged over 60. This suggests that penicillin remains a viable treatment option for elderly patients but may have limited efficacy in paediatric populations.

**Table 2.** Antimicrobial susceptibility profiles of 194 *S*. *pneumoniae* strains

Antibiotic group	Drug name	Antibiotic resistance rate of total isolates					Antibiotic resistance rate of children under 5 years					Antibiotic resistance rate of adults above 60 years				
		N	R	Percentage (%)	S	Percentage (%)	N	R	Percentage (%)	S	Percentage (%)	N	R	Percentage (%)	S	Percentage (%)
Beta-lactams (penicillins)	Penicillin	194	24	12.37	78	40.21	151	19	12.58	52	34.44	28	0	0	20	71.43
	Amoxicillin	194	0	0	116	59.79	85	0	0	85	100	21	0	0	21	100
Beta-lactams (cephalosporins)	Cefotaxime	191	43	22.51	129	67.54	150	36	24.00	99	66.00	26	4	15.38	21	80.77
	Ceftriaxone	135	0	0	135	100	101	0	0	101	100	23	0	0	23	100
Carbapenems	Meropenem	192	46	23.96	60	31.25	150	40	26.67	38	25.33	27	2	7.41	18	66.67
	Ertapenem	192	1	0.52	185	96.35	150	1	0.67	143	95.33	27	0	0	27	100
Fluoroquinolones	Ofloxacin	190	0	0	183	96.32	149	0	0	145	97.32	26	0	0	24	92.31
	Levofloxacin	192	0	0	192	100	150	0	0	150	100.00	27	0	0	27	100
	Moxifloxacin	192	0	0	192	100	150	0	0	150	100	27	0	0	27	100
Tetracyclines	Tetracycline	190	180	94.74	9	4.74	149	144	96.64	5	3.36	27	23	85.19	3	11.11
Macrolides	Erythromycin	191	191	100	0	0	150	150	100	0	0	26	26	100	0	0
	Telithromycin	191	3	1.57	169	88.48	149	3	2.01	131	87.92	27	0	0	24	88.89
Sulphonamides and folate pathway inhibitors	Trimethoprim-sulfamethoxazole	192	124	64.58	40	20.83	150	103	68.67	24	16.00	27	9	33.33	14	51.85
Glycopeptides	Vancomycin	192	0	0	192	100	150	0	0	150	100	27	0	0	27	100
Oxazolidinones	Linezolid	192	0	0	192	100	150	0	0	150	100	27	0	0	27	100
Amphenicols	Chloramphenicol	191	14	7.33	177	92.67	149	10	6.71	139	93.29	27	3	11.11	24	88.89

Penicillin susceptibility breakpoints for *S. pneumoniae* strains: for *S. pneumoniae*, the MIC breakpoints for penicillin susceptibility, as defined by the CLSI 2024 guidelines, vary based on the site of infection. Non-meningitis infections: susceptible (S), MIC ≤0.06 µg ml−1; intermediate (I), MIC=0.12–1 µg ml−1; resistant (R), MIC ≥2 µg ml−1. Meningitis infections: susceptible (S), MIC ≤0.06 µg ml−1; resistant (R), MIC ≥0.12 µg ml−1. Notably, susceptibility testing for ceftriaxone was performed on 135 isolates instead of all 194 due to the availability of testing reagents and clinical relevance considerations.

Cephalosporins demonstrated more variable efficacy. Ceftriaxone showed no resistance across all age groups, making it a highly reliable option for treating *S. pneumoniae* infections. In contrast, cefotaxime resistance was higher in children under 5 years (24.00%) than in elderly patients over 60 years (15.38%), suggesting that its use should be guided by age-specific resistance patterns. Meropenem had an overall resistance rate of 23.96%, with a higher resistance rate in children (26.67%) compared with the elderly (7.41%). In contrast, ertapenem showed negligible resistance (0.52%) and maintained high susceptibility (>95%) across all age groups, making it a more reliable carbapenem option.

### MLST analysis and serotypes of isolated strains

A total of 194 *S*. *pneumoniae* strains were successfully sequenced and underwent rigorous quality assessment, with all strains meeting the criteria for further analysis. Among these, 56 distinct STs were identified, as detailed in Table 3. The most prevalent ST was ST271, accounting for 24.2% of all isolates (47 strains), followed by an unknown ST (14.0%, 28/194) and ST902 (7.7%, 15/194). The remaining STs were found only in a few isolates each (see Table 3). The relatively high proportion of unknown STs suggests the presence of novel or understudied genotypes, highlighting the need for further investigation into their genetic and epidemiological characteristics. Additionally, the considerable genetic diversity observed among low-frequency STs may reflect regional variations in strain distribution or associations with specific resistance profiles.

**Table 3.** The distribution of STs and serotype among 194 *S. pneumoniae* strains

No.	ST	No. of isolates	Percentage	Serotype	No. of isolates	Percentage (%)
1	271	47	24.20%	19F	47	24.23
2	Unknown	28	13.92%	Unknown	6	3.09
				19A	3	1.55
				35B	3	1.55
				06C	2	1.03
				11F	2	1.03
				19F	2	1.03
				8	1	0.52
				34	1	0.52
				36	1	0.52
				06A	1	0.52
				07B	1	0.52
				15B	1	0.52
				16F	1	0.52
				23A	1	0.52
				23F	1	0.52
				3	1	0.52
3	902	15	7.70%	06A	15	7.73
4	90	7	3.60%	06E	6	3.09
				06A	1	0.52
5	876	7	3.60%	14	7	3.61
6	11972	7	3.60%	15A	6	3.09
				19F	1	0.52
7	320	6	3.10%	19A	5	2.58
				19F	1	0.52
8	9114	6	3.10%	Unknown	5	2.58
				19A	1	0.52
9	81	4	2.10%	23F	4	2.06
10	11967	4	2.10%	07B	4	2.06
11	99	3	1.50%	11F	3	1.55
12	230	3	1.50%	23F	2	1.03
				23A	1	0.52
13	3173	3	1.50%	06A	3	1.55
14	63	2	1.00%	15A	2	1.03
15	505	2	1.00%	3	2	1.03
16	2754	2	1.00%	13	2	1.03
17	6555	2	1.00%	15B	2	1.03
18	7752	2	1.00%	35C	2	1.03
19	8250	2	1.00%	16F	2	1.03
20	8738	2	1.00%	06C	2	1.03
21	13026	2	1.00%	06C	2	1.03
22	14702	2	1.00%	34	2	1.03
23	15069	2	1.00%	3	2	1.03
24	15272	2	1.00%	3	2	1.03
25	180	1	0.50%	3	1	0.52
26	342	1	0.50%	23F	1	0.52
27	473	1	0.50%	06B	1	0.52
28	1106	1	0.50%	Unknown	1	0.52
29	1464	1	0.50%	19F	1	0.52
30	1937	1	0.50%	19F	1	0.52
31	2323	1	0.50%	19F	1	0.52
32	3176	1	0.50%	23F	1	0.52
33	3543	1	0.50%	23F	1	0.52
34	4216	1	0.50%	8	1	0.52
35	4537	1	0.50%	06C	1	0.52
36	4560	1	0.50%	34	1	0.52
37	4635	1	0.50%	Unknown	1	0.52
38	5242	1	0.50%	23A	1	0.52
39	6318	1	0.50%	Unknown	1	0.52
40	6327	1	0.50%	35B	1	0.52
41	6339	1	0.50%	06E	1	0.52
42	6340	1	0.50%	06A	1	0.52
43	6542	1	0.50%	16F	1	0.52
44	7751	1	0.50%	35A	1	0.52
45	8526	1	0.50%	06E	1	0.52
46	8589	1	0.50%	15B	1	0.52
47	9246	1	0.50%	28A	1	0.52
48	9396	1	0.50%	23B1	1	0.52
49	9785	1	0.50%	15B	1	0.52
50	9882	1	0.50%	15B	1	0.52
51	11968	1	0.50%	06A	1	0.52
52	12449	1	0.50%	3	1	0.52
53	13646	1	0.50%	23F	1	0.52
54	15630	1	0.50%	06E	1	0.52
55	17171	1	0.50%	23F	1	0.52
56	17173	1	0.50%	23F	1	0.52

Among the 194 *S. pneumoniae* strains analysed in this study, a total of 24 serotypes were identified, with 14 strains failing to be serotyped. Serotype 19F was the predominant serotype, accounting for 27.8% (54/194) of the strains. Other major serotypes included 06A and 23F, representing 11.3% (22/194) and 6.7% (13/194) of the strains, respectively (see Table 3).

### Phylogenetic analysis of isolated strains

**Fig. 1.**
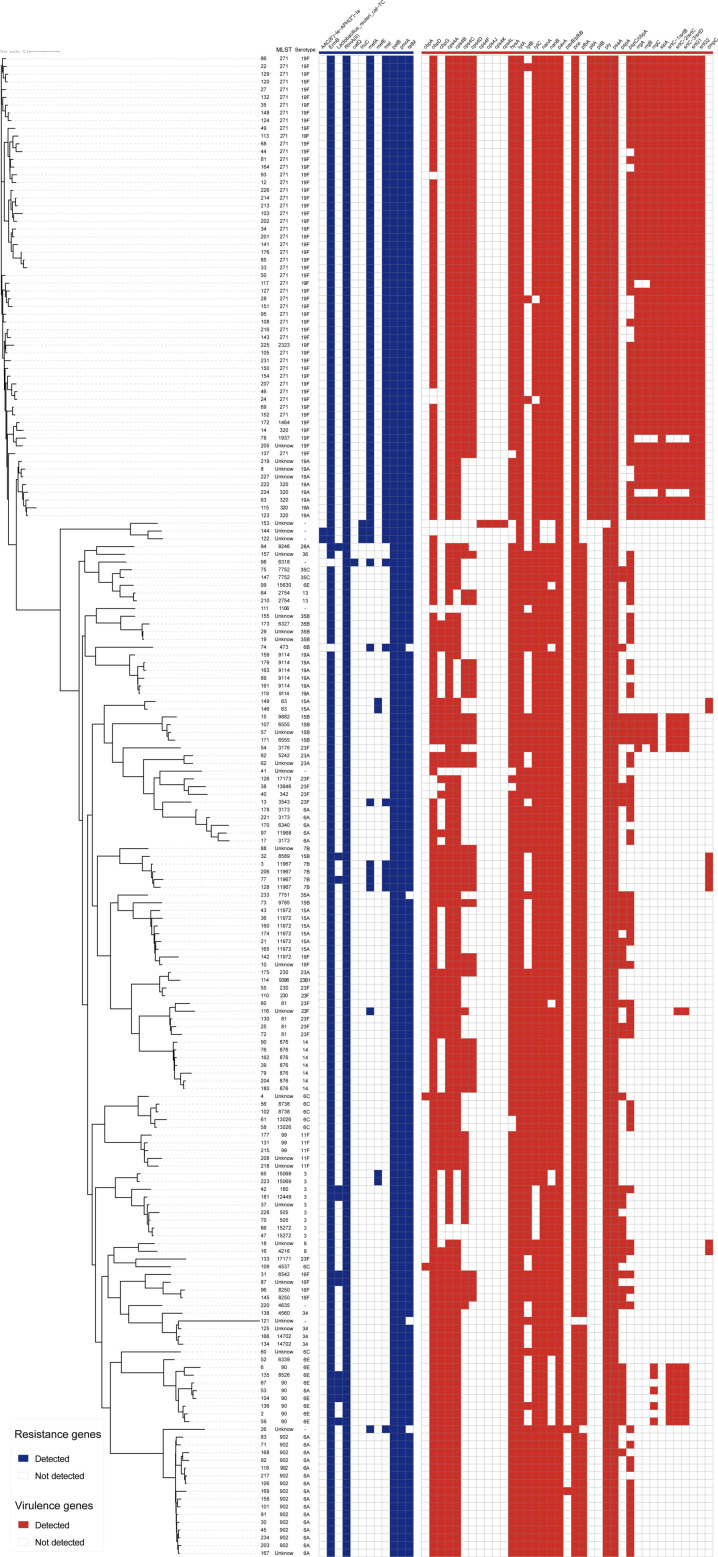
Maximum-likelihood phylogenetic tree of 194 *S*. *pneumoniae* strains based on the core genome, describing the strain number, MLST, serotype, AMR and virulence genes.

The maximum-likelihood phylogenetic tree constructed from WGS data revealed two distinct major clades, each exhibiting unique genetic and serotypic characteristics. The first clade (Fig. 1, top) comprised 61 isolates representing 6 distinct STs, including the prominent ST271 and ST320. These STs are known to be associated with specific serotypes, highlighting a potential link between genetic lineage and serotype distribution. The second clade (Fig. 1, bottom) encompassed a larger and more diverse group of 133 isolates, which were further classified into 50 STs. This clade demonstrated significant genetic diversity, suggesting a broader evolutionary divergence among these strains.

In the MLST analysis, strains sharing the same serotype exhibited a clear clustering pattern, indicating a strong correlation between serotype and genetic lineage. Specifically, ST271, ST320, ST902, ST90 and ST876 were closely associated with serotypes 19F, 19A, 6A, 6E and 14, respectively (Fig. 1). This association underscores the potential role of serotype-specific genetic determinants in the evolution and adaptation of these strains. Notably, serotype 23F, although one of the most common serotypes identified in this study, displayed a remarkable degree of genetic diversity, encompassing a wide range of STs. This finding suggests that serotype 23F may have undergone extensive genetic recombination or horizontal gene transfer, contributing to its high variability.

Regarding antibiotic resistance, the resistance genes *rlmA(II*), *mel*, *patB* and *pmrA* were universally detected across all 194 bacterial strains examined, indicating their widespread presence and potential role in conferring resistance mechanisms. Additionally, the genes *ermB* and *tetM* were identified in the vast majority of the isolates, with detection rates of 99.0% (192/194) and 97.9% (190/194), respectively. These genes are known to mediate resistance to macrolides and tetracyclines, highlighting the prevalence of these resistance mechanisms within the studied population.

All 194 isolates were found to carry the virulence-associated genes *lytA*, *pavA* and *psaA*, which are critical for bacterial survival, colonization and pathogenicity. Furthermore, the majority of the isolates harboured additional virulence genes, including *pce* (98.7%, 192/194), *ply* (98.7%, 192/194), *nanA* (97.9%, 190/194), *cps4A* (96.4%, 187/194), *cbpA* (95.9%, 186/194), *hysA* (95.9%, 187/194), *lytC* (95.4%, 185/194) and *nanB* (90.7%, 176/194), as shown in Fig. 1. These genes play essential roles in various aspects of bacterial virulence, such as immune evasion, adhesion and tissue invasion, further emphasizing the pathogenic potential of these strains. The notably high detection rates of AMR and virulence genes may be associated with extensive antibiotic use in local CAP cases, highlighting a significant antibiotic selection pressure in this region.

### Comparative genomic analysis of ST271 vs. non-ST271 isolates

Through the preceding analysis, we have identified ST271 as the predominant ST in this study. To explore the differences between this ST and others in terms of drug resistance phenotypes, resistance genes and virulence genes, we conducted a detailed analysis.

**Fig. 2.**
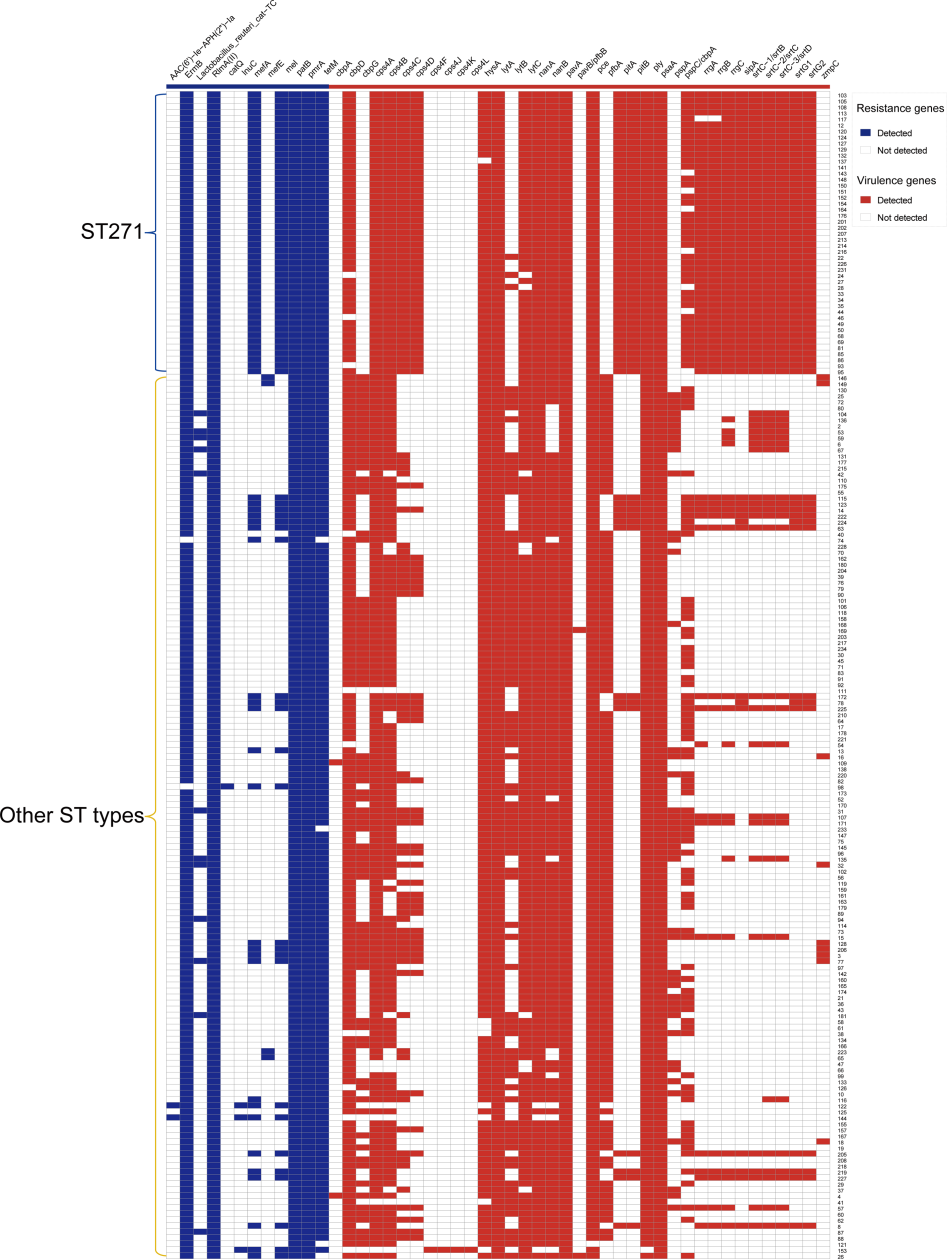
The molecular characteristics of *S. pneumoniae* between the ST271 strains and other STs, including AMR and virulence genes.

Fig. 2 illustrates the distribution of key resistance and virulence genes in ST271 vs. non-ST271 strains. The ST271 strain of *S. pneumoniae* is predominantly associated with serotype 19F, accounting for 87.0% (47/54) of the 19F isolates. Compared with non-ST271 strains, ST271 strains exhibit a higher prevalence of resistance genes such as *metA* and *mel*. Additionally, ST271 strains carry a distinct set of virulence genes, including *cps4C*, *cps4D*, *rrgA*, *rrgB*, *rrgC* and *sipA*. However, an exception is the virulence gene *pfbA*, which is found at a higher frequency in non-ST271 strains. This unique genetic profile highlights the distinct evolutionary and pathogenic characteristics of ST271, particularly its association with serotype 19F and its potential implications for antibiotic resistance and virulence.

**Fig. 3.**
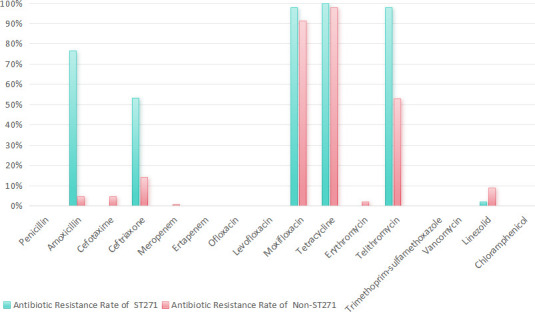
Comparison of AMR rates between ST271 and non-ST271 strains for various antibiotics.

Our research findings reveal that ST271 strains exhibited significantly higher resistance rates than non-ST271 strains for cefotaxime (76.60% vs. 4.76%), meropenem (53.19% vs. 14.29%), trimethoprim-sulfamethoxazole (97.87% vs. 53.06%) and penicillin (21.28% vs. 9.52%) (Fig. 3). Both ST271 and non-ST271 strains exhibited extremely high resistance to erythromycin (100% vs. 97.96%), indicating complete class resistance to macrolides. Similarly, ST271 strains showed notably higher resistance to meropenem (53.19%) than non-ST271 strains (14.29%). Meanwhile, both groups remained fully susceptible to fluoroquinolones, glycopeptides and oxazolidinones.

## Discussion

This study systematically analysed the clinical data, AMR profiles, molecular types and resistance and virulence genes of 194 *S*. *pneumoniae* strains isolated from infected patients, providing valuable insight into *S. pneumoniae* infection in smaller cities and rural areas of Hubei Province.

Over the past few decades, factors such as widespread antibiotic usage and increasing population density have significantly contributed to high colonization and infection rates of *S. pneumoniae* in healthy populations [17]. Our study found that CAP was the most common infection type, accounting for ~90%. The majority of infections occurred in children under 5 years of age (roughly 78%), with male patients making up a larger proportion of cases (about 65%). Furthermore, infections were prevalent during the winter and spring seasons. These findings align with multiregional studies conducted abroad [18] as well as domestic epidemiological surveys on *S. pneumoniae* [19].

In the resistance analysis, we observed that PRSP exhibited significantly higher resistance rates to multiple antibiotics compared with penicillin-sensitive *S. pneumoniae*. This elevated resistance is primarily attributable to the widespread presence and dissemination of AMR genes identified in this study, notably *ermB* and *tetM*, which were detected in 99.0% and 97.9% of isolates, respectively. The *ermB* gene, encoding a ribosomal methylase, serves as the primary mechanism of macrolide resistance, conferring high-level resistance and frequently detected in bacterial populations across mainland China[20,21]. Meanwhile, the *tetM* gene mediates resistance to tetracyclines through ribosomal protection mechanisms [22]. Since the 1990s, empirical antibiotic therapy for suspected *S. pneumoniae* infections has increasingly favoured macrolides due to their efficacy against *Mycoplasma pneumoniae* and their established safety profile in paediatric infectious diseases. However, the widespread and often inappropriate use of these antibiotics has driven a continuous rise in resistance to these drugs [23]. The high detection rates and co-occurrence of these resistance determinants clearly highlight their pivotal roles in driving multidrug-resistant phenotypes among PRSP isolates, posing significant challenges for clinical treatment and emphasizing the critical need for enhanced antibiotic stewardship efforts.

According to the MLST results, 56 distinct STs were identified, with ST271 being the most prevalent, representing 24.2% of all isolates. This finding underscores the high local prevalence of ST271, which aligns with reports of its widespread distribution across Asia [24,25]. Notably, the ST271 strain of *S. pneumoniae* is predominantly associated with serotype 19F. The predominant serotype 19F (associated with ST271) is covered by the PCV13 [26], indicating the potential efficacy of current vaccination strategies in reducing infections by this highly resistant strain. Nevertheless, the presence of multiple serotypes not covered by existing vaccines highlights the need for continuous surveillance and consideration of broader-coverage pneumococcal vaccines in the future.

Compared with non-ST271 strains, ST271 strains exhibit a higher prevalence of resistance genes such as *metA* and *mel*, as well as a distinct set of virulence genes. It has been reported that the induction mechanism of *mel* resembles the transcriptional attenuation observed in the *ermK* methylase gene and *tetM* gene, wherein antibiotic-bound ribosomes stall during the translation of a leader peptide located upstream of the structural gene [27]. However, an exception is the virulence gene *pfbA*, which is found at a higher frequency in non-ST271 strains. This unique genetic profile highlights the distinct evolutionary and pathogenic characteristics of ST271, particularly its strong association with serotype 19F and its potential implications for antibiotic resistance and virulence. Furthermore, the identification of a large number of low-frequency and unknown STs indicates significant genetic diversity among the strains, suggesting the potential presence of novel genotypes that warrant further investigation. These findings collectively emphasize the importance of monitoring ST271 and other emerging genotypes [28], as their genetic makeup may influence their transmission dynamics, resistance patterns and pathogenic potential. Understanding these factors is critical for guiding targeted interventions, such as vaccine development and antibiotic stewardship programmes, to mitigate the public health impact of *S. pneumoniae* infections.

The ST271 of *S. pneumoniae* exhibited significantly higher resistance to *β*-lactams (cefotaxime), carbapenems (meropenem), tetracyclines and sulphonamides compared with non-ST271 strains. Importantly, both ST strains remained susceptible to fluoroquinolones, glycopeptides and oxazolidinones. These results underscore the critical importance of judicious antibiotic selection for ST271-associated infections. When managing IPD, clinicians must exercise particular caution in antibiotic choice, with special consideration for paediatric cases. Ceftriaxone, demonstrating consistently high susceptibility rates, emerges as a preferable empirical option. Additionally, these findings strongly support the implementation of reinforced antibiotic stewardship initiatives at the institutional level to curb the dissemination of these resistant clones.

This study has several limitations. Firstly, as data were derived from a single-centre analysis over 1 year, the results might not fully reflect the broader epidemiological dynamics in other regions of China or across multiple years. Future multi-centre, longitudinal studies are needed to validate these findings. Secondly, the absence of clinical outcomes associated with specific resistance profiles and serotypes limits the ability to directly correlate microbial characteristics with treatment outcomes and patient prognosis. Finally, our study did not evaluate the actual vaccination status of the patients or the regional vaccine coverage rate, restricting the interpretation of vaccine efficacy on the observed serotype distribution. Future research incorporating vaccination data and clinical outcomes is warranted.

## Conclusion

In conclusion, this study highlights a significant burden of invasive *S. pneumoniae* infections, predominantly affecting children under five, with notable peaks during winter and spring. ST271, predominantly associated with serotype 19F, exhibited significantly higher antibiotic resistance rates compared with other strains, indicating the necessity of tailored antibiotic strategies and robust local antibiotic stewardship programmes. The widespread presence of resistance and virulence genes underscores the evolutionary adaptability of *S. pneumoniae*, emphasizing the importance of continuous genetic surveillance. The current pneumococcal vaccination (PCV13) coverage of the predominant serotype provides a favourable outlook for disease control; however, monitoring non-vaccine serotypes remains critical. Future research should integrate clinical outcomes, longitudinal surveillance and regional vaccination coverage assessments to comprehensively inform public health interventions.

## Abbreviations

AMR: antimicrobial resistance
CAP: community-acquired pneumonia
CLSI: Clinical and Laboratory Standards Institute
HAP: hospital-acquired pneumonia
IPD: invasive pneumococcal disease
MLST: multilocus sequence typing
PCV13: 13-valent pneumococcal conjugate vaccine
PRSP: penicillin-resistant *Streptococcus pneumoniae*
ST: sequence type
WGS: whole-genome sequencing
